# Spectrum Analysis of Thermally Driven Curvature Inversion in Strained Graphene Ripples for Energy Conversion Applications via Molecular Dynamics

**DOI:** 10.3390/nano15171332

**Published:** 2025-08-29

**Authors:** James M. Mangum, Md R. Kabir, Tamzeed B. Amin, Syed M. Rahman, Ashaduzzaman, Paul M. Thibado

**Affiliations:** 1 Department of Physics, University of Arkansas, Fayetteville, AR 72701, USA; jmmangum@uark.edu (J.M.M.); tbamin@uark.edu (T.B.A.); 2 Materials Science and Engineering Program, University of Arkansas, Fayetteville, AR 72701, USA; kabir@uark.edu (M.R.K.); sr096@uark.edu (S.M.R.); a004@uark.edu (A.)

**Keywords:** graphene, ripples, thermal fluctuations, strain, bistability, molecular dynamics, energy harvesting

## Abstract

The extraordinary mechanical flexibility, high electrical conductivity, and nanoscale instability of freestanding graphene make it an excellent candidate for vibration energy harvesting. When freestanding graphene is stretched taut and subject to external forces, it will vibrate like a drum head. Its vibrations occur at a fundamental frequency along with higher-order harmonics. Alternatively, when freestanding graphene is compressed, it will arch slightly out of the plane or buckle under the load. Remaining flat under compression would be energetically too costly compared to simple bond rotations. Buckling up or down, also known as ripple formation, naturally creates a bistable situation. When the compressed system vibrates between its two low-energy states, it must pass through the high-energy middle. The greater the compression, the higher the energy barrier. The system can still oscillate but the frequency will drop far below the fundamental drum-head frequency. The low frequencies combined with the large-scale movement and the large number of atoms coherently moving are key factors addressed in this study. Ten ripples with increasing compressive strain were built, and each was studied at five different temperatures. Increasing the temperature has a similar effect as increasing the compressive strain. Analysis of the average time between curvature inversion events allowed us to quantify the energy barrier height. When the low-frequency bistable data were time-averaged, the authors found that the velocity distribution shifts from the expected Gaussian to a heavy-tailed Cauchy (Lorentzian) distribution, which is important for energy harvesting applications.

## 1. Introduction

Recent advances in ultralow-power-consuming electronics have led to significant improvements in modern sensor systems. Devices draw nanowatts in standby mode and are programmed to spend very little time in active mode [1]. As a result, new low-power systems can potentially draw all their energy requirements from ambient sources, eliminating the need for battery replacement and enabling autonomous operation in remote and difficult-to-access locations.

Many ambient power sources are being explored for energy harvesting to run micro-scale systems such as mechanical vibrations, solar radiation, and thermal gradients [2]. Among these power sources, vibration-based mechanical energy is omnipresent but often low power in nature, while solar and thermal energy can be intermittent [3]. Energy harvesting from multiple sources may enable the continuous operation of low-power electronics.

Initial designs for mechanical energy harvesting used a varying capacitance system as an electrostatic generator [4]. Subsequent advancements demonstrated the viability of microelectromechanical systems (MEMSs) utilizing electrostatic and piezoelectric properties to harvest energy from environmental vibrations [5,6].

A fundamental consideration in the design of mechanical energy harvesters is the flexural rigidity of the vibrating material, since it increases with the cube of its thickness. This limitation has led researchers to consider using ultrathin suspended graphene. For example, taut graphene membranes were used to fabricate high-frequency resonators [7]. Applying a bias voltage between suspended graphene and a fixed plate was used to build a tuneable capacitor [8]. Additionally, when suspended over arrays of nanopillars, graphene exhibits unique nonlinearities in its electronic density of states driven by enhanced electron–electron interactions in the freestanding regions [9].

Experimentalists found that suspended graphene is not perfectly flat. It naturally forms nanoscale ripples and corrugations due to self-compression and intrinsic thermal fluctuations [10,11,12]. Out-of-plane deformations are fundamental to the existence of a freestanding atomic membrane [13].

Theoretical studies also attribute ripple formation to thermal fluctuations and electron–phonon coupling within the two-dimensional lattice [14,15]. These mechanisms modulate the elastic energy and relate ripple morphology to local charge density variations [16]. Notably, strong electron–phonon interactions have been predicted to favor a rippled ground state, suggesting a potential phase transition in structural configuration [17]. This prediction is supported by both computational and theoretical models. Density functional theory calculations confirm that graphene possesses exceptionally low bending rigidity, making it energetically favorable for the material to adopt out-of-plane deformations in response to internal fluctuations [18]. In parallel, magnetotransport models further demonstrate that in-plane magnetic fields interacting with ripple-induced curvature produce anisotropic magnetoresistance, reinforcing the view that graphene ripples are stable structural features with a functional electronic impact [19]. Such rippling in suspended graphene is now understood to stabilize 2D materials at finite temperatures, countering the classical prediction of instability [20].

As a graphene membrane absorbs ambient thermal energy, stochastic transitions can occur. Ripples can invert their curvature through thermally activated barrier crossing, in line with Kramers’ escape theory [21]. The fluctuations modulate charge transport across the surface, giving rise to low-frequency resistance variations and 1/f noise. Additionally, out-of-plane distortions are not isolated events but spatially correlated, allowing curvature changes in one region to influence distant areas. The interconnected ripple dynamics can be driven by thermal motion and mechanical coupling [22,23].

Curvature inversion is not confined to nanoscale systems. In MEMS actuators and buckled elastic shells, geometric instabilities are intentionally embedded to trigger rapid transitions between mechanically stable configurations. These systems store elastic energy in preloaded states and release it when a critical threshold is surpassed, allowing the structure to snap into a new equilibrium. This dynamic switching across an energy barrier enables amplified mechanical responses, outperforming linear counterparts in terms of range, speed, and efficiency [24,25,26].

Barrier-crossing dynamics offer a compelling route to harvest energy from low-frequency, broadband vibrations. By engineering bistable systems with nonlinear restoring forces, snap-through transitions inject sharp bursts of kinetic energy into the response. These spikes in velocity amplify power output without relying on resonance. Unlike linear designs constrained to narrow frequency bands, bistable harvesters harness geometric instability to convert ambient motion into useful electrical energy across a wider operational spectrum [27].

Experiments reveal that bistable energy harvesters reliably convert ambient vibrations into electrical power through barrier-crossing events. When driven beyond a critical displacement, the system undergoes rapid transitions between stable states, producing sharp velocity surges that amplify power output. These snap-through dynamics persist across a range of frequencies, demonstrating resilience to both low-frequency and broadband excitation [28].

In a similar way, compressed graphene exhibits spontaneous curvature inversions. These transitions are discrete and collective, governed by internal factors such as lattice coupling and geometric constraints [29,30]. Recent simulations confirm that these states are robust across different system sizes and follow dynamic scaling relationships [31,32]. As compression increases, the system crosses critical thresholds in radius and temperature, resulting in sharp, controllable shifts in curvature [33]. Compressed graphene behaves as a multistable mechanical system capable of intrinsic curvature control.

Atomic force microscopy experiments have directly observed snap-through instabilities and measured adhesion energies with nanoscale precision [34,35]. These findings have motivated theoretical studies on harnessing the inherent bistability of compressed graphene for nonlinear energy harvesting, especially at low frequencies [36]. Analytical models identify well-defined thresholds for snap-through transitions, confirming graphene’s suitability for efficient and tunable mechanical-to-electrical energy conversion [37].

Finally, the surface chemistry of graphene can also be impacted by the dynamics of surface atoms. Often, static structural models are used, but dynamics can play a critical role in dictating the overall chemical reactivity by directly impacting the adsorption strength of reactants, the kinetics of elementary reaction steps, and, ultimately, the selectivity and efficiency of catalytic processes. Our work within this Special Issue contributes to a more comprehensive understanding of surface chemistry by showcasing specific surface atom dynamics, thereby potentially bridging the gap between static structural approaches and the dynamic complexity of systems.

In this study, we present the frequency response for graphene curvature inversion as a function of strain and temperature using molecular dynamics simulations. Ten ripples with increasing compressive strain were built, and each was studied at five different temperatures.

## 2. Materials and Methods

**Figure 1:**
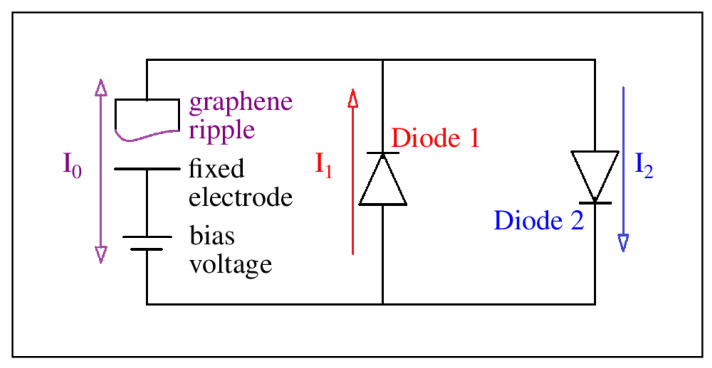
A schematic of a graphene energy-harvesting circuit that includes a variable capacitor consisting of a fixed electrode below a suspended sheet of graphene, a rechargeable battery, and two diodes for rectification.

Our motivation for conducting this study is best illustrated using the schematic of a graphene energy-harvesting circuit shown in Figure 1. The circuit consisted of a graphene-based variable capacitor in the left branch, along with a DC rechargeable bias voltage source. The variable capacitor was formed from a fixed electrode positioned below a suspended sheet of graphene. As the graphene vibrated, due to external forces, the distance from the fixed electrode changed over time. As the capacitance changed in time, charges were driven on and off the capacitor due to the fixed bias voltage. When positive charges flowed clockwise, they passed through diode 2 and produced current $I_{2}$. When positive charges flowed counterclockwise, they moved against the bias voltage to recharge it, passed through diode 1, and produced current $I_{1}$. The source of power came from the forces moving the graphene. Two storage capacitors could be used to harvest energy for later use. In a previous study, we critically analyzed the fundamental mechanisms behind ripple curvature inversion, and those results can be found in [38].

To predict the behavior of the graphene energy-harvesting (GEH) circuit, we first wanted to understand the frequency response of the freestanding graphene. If the frequencies were too high, for example, the circuit would not be able to respond to the changes in capacitance. We constructed a series of 10 graphene ripples with consecutively higher compressive strain and numbered them from 1 to 10 for easier reference. Each ripple had about 10,000 carbon atoms. Higher ripple numbers corresponded to higher compressive strain. To create a ripple, we started by generating a flat, hexagonal array of carbon atoms with a bond length of 1.4 Å. The position of every atom was multiplied by a factor $\left(1-\epsilon_{i}\right)$, where $\epsilon_{i}$ is the initial compressive strain given by

(1) $$\epsilon_{i}=\frac{L_{0}-L}{L_{0}},$$

where $L_{0}$ is the original length of the graphene sheet and *L* is the new compressed length of the graphene sheet. Once the flat lattice had been uniformly compressed, a vertical component was added to every atom within a radius of r=75 Å, using

(2) $$z\left(x,y\right)=h_{0}\;cos\left[\frac{\pi }{2}\frac{1}{75}\sqrt{x^{2}+y^{2}}\right],$$

where $h_{0}$ is chosen such that the average bond length returns to 1.4 Å, which aids the simulation. An annulus spanning from radius 75 Å to 90 Å remains flat and frozen.

**Figure 2:**
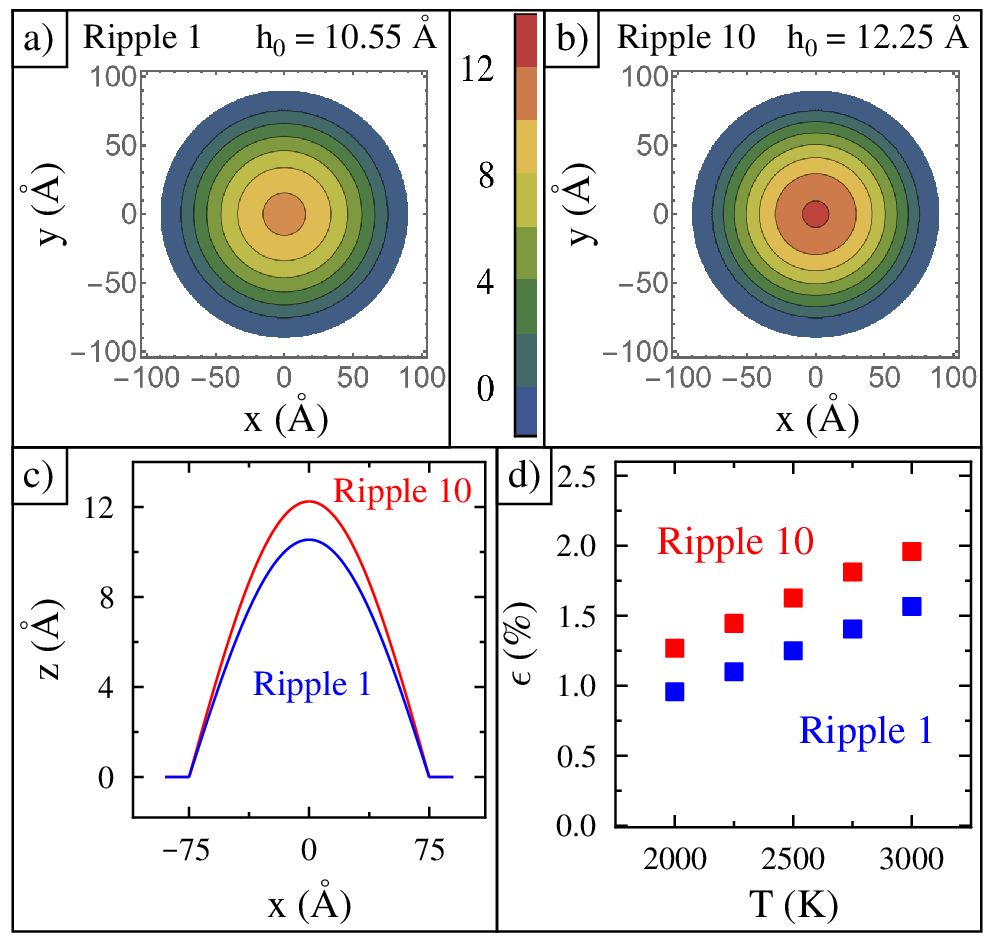
The structure and strain of the graphene ripples. A color image of (**a**) ripple 1 and (**b**) ripple 10. The height of the ripple is shown in angstroms using the color scale bar drawn between the top two panels. (**c**) Cross-sectional line profiles of ripple 1 and 10. (**d**) Compressive strains of ripples 1 and 10 at the five temperatures used in this study.

A top-view color scale image of ripple 1 is shown in Figure 2a. It shows a gently curved surface with an initial central height of 10.55 Å. The height drops off as you go outward from the center, as represented by the concentric circular contour lines. Ripple 1 has the lowest compressive strain of any ripple simulated in this study. A top-view color scale image of ripple 10 is shown in Figure 2b. It is the tallest ripple with a central height of 12.25 Å. Ripple 10 has the highest compressive strain of any ripple in this study. A cross-sectional line profile of ripples 1 and 10 along the y=0 axis is shown in Figure 2c.

Once the cosine-shaped ripples were formed, they were input into the molecular dynamics simulation LAMMPS. We used the AIREBO-C inter-atomic potential and applied a cutoff distance of 3 standard deviations. The computation time step was 1 femtosecond. The temperature and force on all atoms in the annulus between radius 75 Å and 90 Å were set to zero, while the temperature of the elevated ripple inside a radius of 75 Å was maintained using a Nose–Hoover thermostat set to 2000, 2250, 2500, 2750, or 3000 K. The simulations were run at elevated temperatures to speed up the dynamics.

The coordinates of all the atoms were tracked every picosecond for the duration of the simulation, which was 40 ns. The first two nanoseconds of the simulation were discarded. Once each ripple reached its equilibrium shape, we measured the actual length of the graphene membrane to determine the strain, which is shown in Figure 2d. This plot shows only ripples 1 and 10 for each of the five temperatures studied. The strain increases linearly with temperature. Ripples 2 to 9 have strains in between 1 and 10. Ripple 1 has the lowest strain, which is 0.9% at 2000 K. The highest strain studied is 1.9% for ripple 10 at 3000 K.

## 3. Results and Discussion

**Figure 3:**
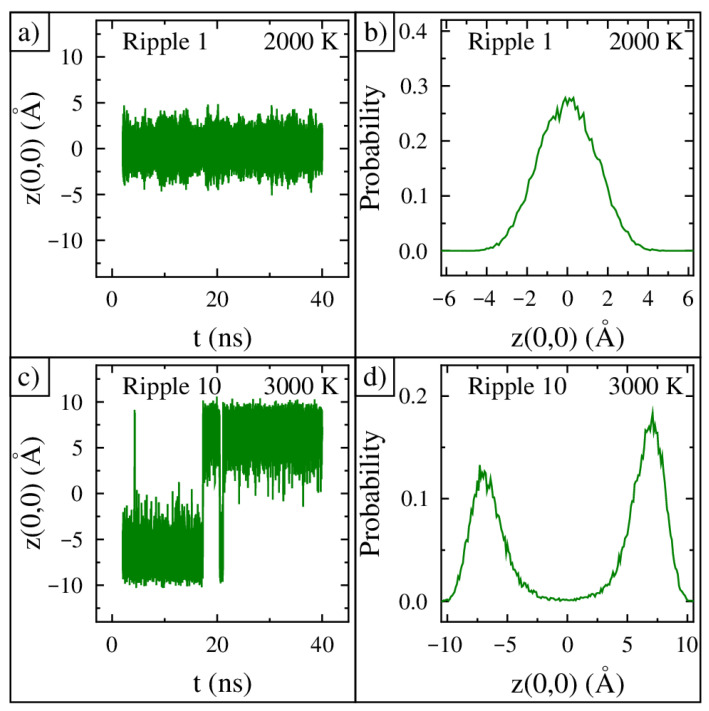
Ripple center dynamics and probability. (**a**) Tracking the center position of ripple 1 at 2000 K in time. (**b**) The probability distribution for the position of ripple 1 held at 2000 K. (**c**) Tracking the center position of ripple 10 at 3000 K in time. (**d**) The probability distribution for the position of ripple 10 held at 3000 K.

The time evolution of the center of ripple 1 at 2000 K from 2 ns to 40 ns is shown in Figure 3a. The center moves at a very high frequency and oscillates at least 5 Å from minimum to maximum, and its movement is centered at a height of zero (the defined location of the frozen annulus). The probability distribution for the vertical position of the center of ripple 1 at 2000 K is shown in Figure 3b. The ripple has the highest probability of being at a height of zero, and the probability falls off uniformly from this height. To find the probability distribution, we first calculated the average height of the 13 central atoms in angstroms. This average value was calculated every picosecond, which produced 38,000 data points with values between −10 and +10 angstroms. Next, using a bin size of 0.1 angstroms, a histogram was built. Finally, the area under the histogram was rescaled to one.

The time evolution of the center of ripple 10 at 3000 K from 2 ns to 40 ns is shown in Figure 3c. In the beginning, the center of the ripple is at position −7 Å on average. At 5 ns, its position jumps to +9 Å but only for an instant. In total, five flips across the zero position occur in this simulation. The probability distribution of the vertical position of the center of ripple 10 at 3000 K is shown in Figure 3d. The distribution has two clear peaks centered at −7 Å and +7 Å. Furthermore, the ripple has a negligible probability of being at a height of zero. Given the large distances moved by the center and the amount of time held at each position, each flip corresponds to ripple 10 inverting its curvature between concave and convex shapes.

To provide more insight into the value of the bimodal distribution shown in Figure 3d, let us reconsider our energy-harvesting circuit shown in Figure 1. The graphene ripple is above a fixed electrode, and together they form a capacitor with capacitance C(t)=ϵA/d(t), where ϵ is the permittivity, *A* is the area of the graphene, and d(t) is the distance between the two electrodes that changes in time [39]. The charge on the graphene ripple is given by $q\left(t\right)=C\left(t\right)V_{0}$, where $V_{0}$ is the fixed bias voltage. For a given graphene oscillation frequency, *f*, we can write $C\left(t\right)=C_{00}+C_{0}$ sin(2πft), where $C_{00}$ is the offset capacitance and $C_{0}$ is the amplitude of the capacitance variation. The instantaneous current is given by $\dot{q}=2\pi fC_{0}V_{0}$ cos(2πft), and, therefore, the average current passing through the diode is given by $I_{ave}=2fC_{0}V_{0}$. Current variations in time can easily yield nanoamps with a single ripple [38].

**Figure 4:**
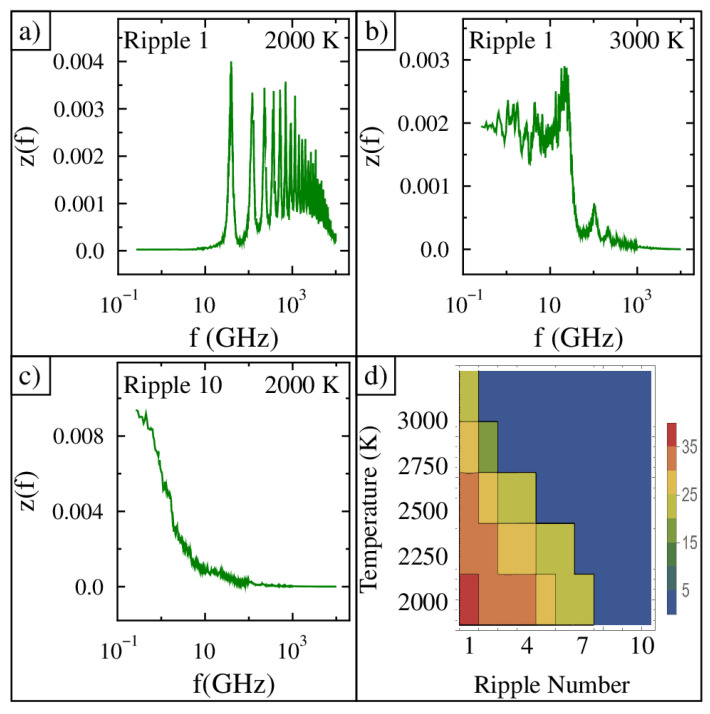
The frequency response for the vertical motion of (**a**) ripple 1 at 2000 K, (**b**) ripple 1 at 3000 K, and (**c**) ripple 10 at 2000 K. (**d**) The frequency of the highest-amplitude peak plotted versus both ripple number (strain) and simulation temperature. The frequency of the ripple is shown in GHz using the color scale bar drawn to the right of the panel.

The spectral response of ripple 1 at 2000 K is shown in Figure 4a. There is no low-frequency response, and there is a series of narrow tall peaks at higher frequencies. The lowest-frequency peak is at 40 GHz and the ones above it are integer multiples of 40 GHz. The spectral response of ripple 1 at 3000 K is shown in Figure 4b. Here, we find a few peaks above 40 GHz, but they are a lot smaller in amplitude and wider. More importantly, a larger broader response now occurs below 40 GHz. The spectral response of ripple 10 at 2000 K is shown in Figure 4c. The highest amplitude now occurs at the lowest frequency, the amplitude significantly decreases with increasing frequency, and no peaks are present in this spectrum. The frequency of the highest amplitude peak for all 10 ripples at all five temperatures is shown in Figure 4d. A sharp drop in the peak frequency occurs systematically as the ripple number increases and the temperature increases.

Spectral analysis reveals that ripples with lower strain and lower temperature exhibit high-frequency oscillations with distinct peaks. In addition, the probability distribution shows the ripple resides in a single potential energy well. At low temperatures, ripple 1 behaves like a tightly stretched membrane, oscillating rapidly about height equals zero. In contrast, ripples with more strain or at elevated temperatures shift to dramatically lower frequencies (sub-fundamental) with a continuous spectrum. Ripple 10, for example, vibrates more slowly and intermittently switches between two locations away from a height of zero. As the simulation temperature increases, the bond length increases. As a result of thermal expansion, the strain increases and alters the vibration response, similar to increases in the applied strain.

From the above results, it is clear that the system can switch from fundamental mode vibration to energy barrier-crossing vibration by increasing the applied strain or the lattice temperature. It is possible to quantify the energy barrier present in each ripple by analyzing the average time spent between curvature inversion events. The energy barrier is due to the compressive strain through the expression

(3) $$\Delta U=\alpha \;\epsilon^{2},$$

where α determines how the energy barrier increases with strain [36]. Furthermore, the time between inversion events is dictated by Kramer’s rate:

(4) $$<t>\;\propto \;e^{\frac{\Delta U}{k_{B}T}}.$$

**Figure 5:**
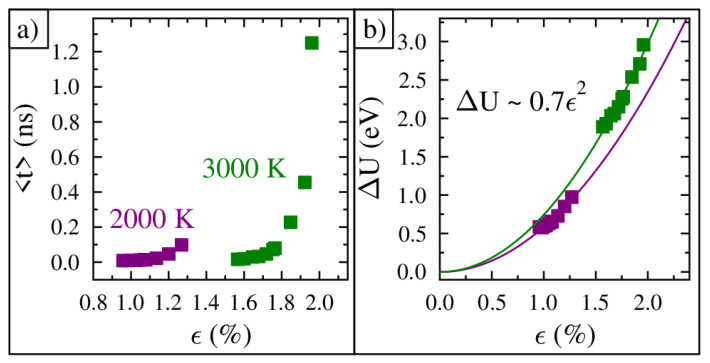
The time between curvature inversion events and the energy barrier height. (**a**) The average time between curvature inversions versus applied strain (ripple number) for two simulation temperatures. (**b**) Energy barrier height versus applied strain (ripple number) for two simulation temperatures.

A plot of the average time between inversion events versus the measured strain (ripple number) for two temperatures is shown in Figure 5a. Notice how the average time increases exponentially as the strain increases. A plot of the calculated barrier height versus applied strain (ripple number) for two temperatures is shown in Figure 5b. The solid lines through the data are a best fit for Equation (3) [36]. Notice that increasing the temperature increases the barrier height, which is due to the bond length increasing.

Optimizing graphene ripples for the purpose of energy harvesting requires high enough compressive strain for the emergence of bistability but low enough compressive strain that the ripple does not become stuck on one side for too long a time period [36].

When the ripple temperature is set, of course the velocity distribution is fixed to be a Gaussian. However, when a system hops between two stable configurations, it can impact the time-averaged velocity distribution. In our previous study, we observed that a Gaussian can be transformed into a Cauchy (Lorentzian) distribution, for example, in [40]. This is interesting and relevant because energy can be harvested from non-Gaussian noise [41]. In addition, ultra-sensitive sensors have recently been fabricated from buckled graphene [42].

**Figure 6:**
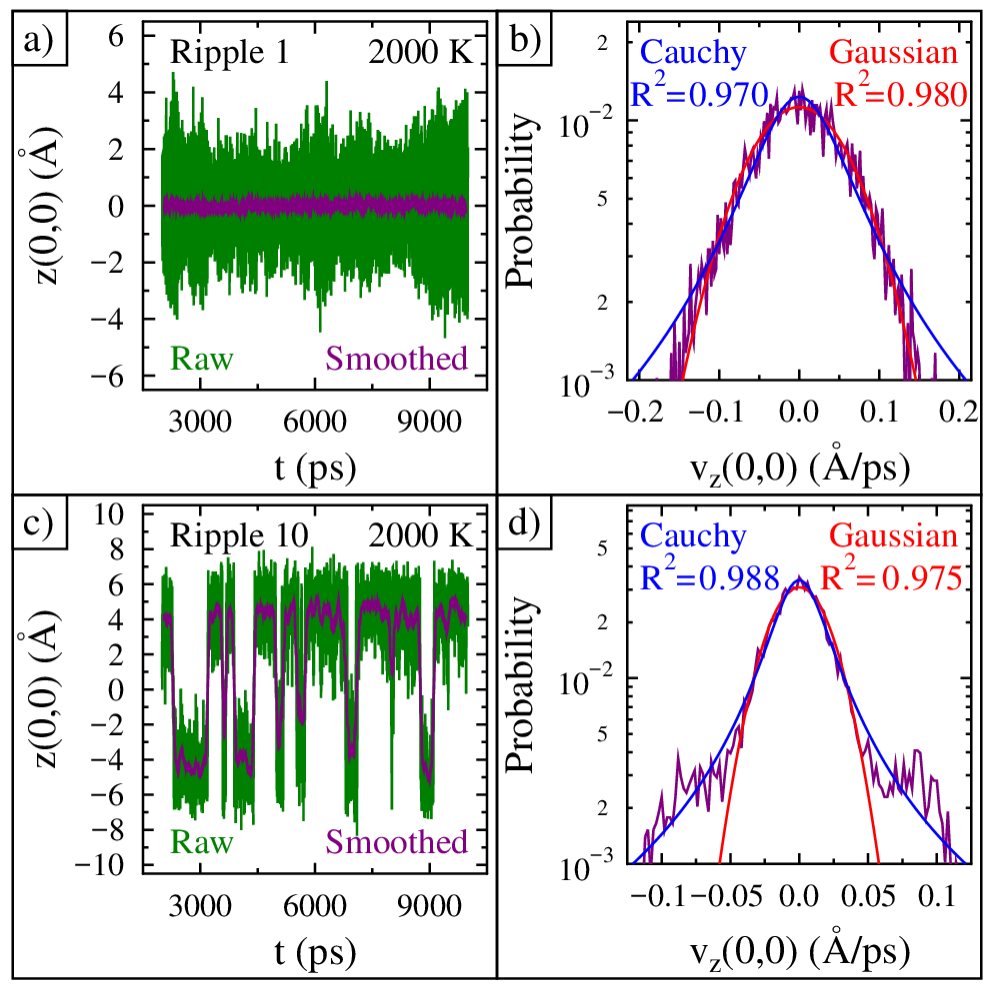
Analyzing the moving-averaged (smoothed) velocity distribution of ripples. (**a**) Raw and smoothed central height of ripple 1 at 2000 K. (**b**) Velocity distribution for raw and smoothed ripple 1 data at 2000 K. (**c**) Raw and smoothed central height of ripple 10 at 3000 K. (**d**) Velocity distribution for raw and smoothed ripple 10 at 3000 K.

The time evolution of the center of ripple 1 at 2000 K over the course of 8 ns is shown in Figure 6a. The green curve shows the raw central position data, while the purple curve is the time-averaged data using every 100 data points. We time-averaged the movement because any electrical circuit sensitive to the ripple curvature inversion events cannot respond to the higher frequencies of the single carbon atom oscillations. The velocity probability distribution for the smoothed ripple 1 data at 2000 K is shown in Figure 6b. It closely follows a Gaussian profile. A best-fit Cauchy distribution is also shown, but the fit is poorer at the highest speeds.

The time evolution of the center of ripple 10 at 2000 K is shown in Figure 6c. The green curve shows the raw central position data, while the purple curve is the time-averaged data taken using every 100 data points. The velocity probability distribution for the smoothed ripple 10 data at 2000 K is shown in Figure 6d. The two fits capture the data well for low speeds, but the Cauchy distribution has the best fit at higher speeds. The Cauchy distribution is special in that it is a form of non-equilibrium noise and reveals that the highest velocity movements occur much more often than expected. We time-averaged the movement because any electrical circuit sensitive to ripple movement cannot respond to the higher frequencies. Furthermore, during this movement, all the carbon atoms move coherently together from a convex to a concave ripple curvature. To recap, after smoothing, the Cauchy distribution fits the bimodal graphene movement best, especially at higher velocities. This is due to the entire graphene ripple inverting its curvature, and in the process, the carbon atoms must undergo a large displacement, which leads to a large velocity.

## 4. Conclusions

In summary, the authors used molecular dynamics simulations to study the out-of-plane dynamics of suspended graphene ripples. The authors built ten graphene ripples with varying compressive strain. Each ripple started with a cosine shape, which aided the simulation process. All ten ripples were run at five different temperatures between 2000 and 3000 K. After stabilization, the compressive strain spanned from 0.9% to 1.9%.

Ripple movement was tracked for 40 ns. For low strain and low temperatures, the ripples oscillated symmetrically at about a height of zero. As the applied strain increased or the simulation temperature increased, the dynamics dramatically shifted to bistable movement away from zero height. In this case, the ripple center spent most of its time at a height of either +7 Å or −7 Å. Furthermore, the position probability distribution showed that very little time was spent at a height of zero.

Spectrum analysis of the movement showed that low-strain and low-temperature ripples oscillated with the highest frequencies. The spectrum had many narrow peaks starting as low as 40 GHz. All higher frequency peaks were integer multiples of this fundamental frequency. As the applied strain increased or the simulation temperature increased, the graphene abruptly transitioned to having only sub-40 GHz frequency oscillations. A sharp transition line occurred in the plane of temperature and strain.

For the bistable ripples, the authors analyzed the average time between transitions to determine the energy barrier height as a function of both temperature and strain. The authors also showed that when the bistable movement was time-averaged, the velocity distribution changed from the expected Gaussian to a heavy-tailed Cauchy (Lorentzian) distribution.

## Data Availability

The original contributions presented in this study are included in this article. Further inquiries can be directed to the corresponding author.
